# Factors Associated with Positive SARS-CoV-2 Test Results in Outpatient Health Facilities and Emergency Departments Among Children and Adolescents Aged <18 Years — Mississippi, September–November 2020

**DOI:** 10.15585/mmwr.mm6950e3

**Published:** 2020-12-18

**Authors:** Charlotte V. Hobbs, Lora M. Martin, Sara S. Kim, Brian M. Kirmse, Lisa Haynie, Sarah McGraw, Paul Byers, Kathryn G. Taylor, Manish M. Patel, Brendan Flannery, CDC COVID-19 Response Team, CDC COVID-19 Response Team, Carmen S. Arriola, Eric P. Griggs, Ashley K. Simon, Meagan E. Stephenson

**Affiliations:** ^1^Children’s of Mississippi, University of Mississippi Medical Center, Jackson, Mississippi; ^2^School of Nursing, University of Mississippi Medical Center, Jackson, Mississippi; ^3^CDC COVID-19 Response Team; ^4^Mississippi State Department of Health, Jackson, Mississippi.; CDC; CDC; CDC; CDC.

As of December 14, 2020, children and adolescents aged <18 years have accounted for 10.2% of coronavirus disease 2019 (COVID-19) cases reported in the United States.* Mitigation strategies to prevent infection with SARS-CoV-2, the virus that causes COVID-19, among persons of all ages, are important for pandemic control. Characterization of risk factors for SARS-CoV-2 infection among children and adolescents can inform efforts by parents, school and program administrators, and public health officials to reduce SARS-CoV-2 transmission. To assess school, community, and close contact exposures associated with pediatric COVID-19, a case-control study was conducted to compare exposures reported by parents or guardians of children and adolescents aged <18 years with SARS-CoV-2 infection confirmed by reverse transcription–polymerase chain reaction (RT-PCR) testing (case-patients) with exposures reported among those who received negative SARS-CoV-2 RT-PCR test results (control participants). Among 397 children and adolescents investigated, in-person school or child care attendance ≤14 days before the SARS-CoV-2 test was reported for 62% of case-patients and 68% of control participants and was not associated with a positive SARS-CoV-2 test result (adjusted odds ratio [aOR] = 0.8, 95% confidence interval [CI] = 0.5–1.3). Among 236 children aged ≥2 years who attended child care or school during the 2 weeks before SARS-CoV-2 testing, parents of 64% of case-patients and 76% of control participants reported that their child and all staff members wore masks inside the facility (aOR = 0.4, 95% CI = 0.2–0.8). In the 2 weeks preceding SARS-CoV-2 testing, case-patients were more likely to have had close contact with a person with known COVID-19 (aOR = 3.2, 95% CI = 2.0–5.0), have attended gatherings^†^ with persons outside their household, including social functions (aOR = 2.4, 95% CI = 1.1–5.5) or activities with other children (aOR = 3.3, 95% CI = 1.3–8.4), or have had visitors in the home (aOR = 1.9, 95% CI = 1.2–2.9) than were control participants. Close contacts with persons with COVID-19 and gatherings contribute to SARS-CoV-2 infections in children and adolescents. Consistent use of masks, social distancing, isolation of infected persons, and quarantine of those who are exposed to the virus continue to be important to prevent COVID-19 spread.

This investigation included children and adolescents aged <18 years who received testing for presence of SARS-CoV-2 in nasopharyngeal swab specimens by RT-PCR at outpatient testing health care centers (including drive-up testing locations) or emergency departments associated with the University of Mississippi Medical Center (UMMC) during September 1–November 5, 2020 (*1*). A COVID-19 case was confirmed by a positive SARS-CoV-2 RT-PCR test result. After excluding inconclusive RT-PCR results, lists of children and adolescents with an electronic medical record of a SARS-CoV-2 test within the study period were randomly ordered by laboratory result. Children with negative SARS-CoV-2 RT-PCR test results were frequency matched to the number of case-patients enrolled by age group (0–3, 4–8, 9–14 and 15–17 years), sex, and test date interval (September 1–24, September 22–October 18, and October 14–November 5, 2020),^§^ with a target sample size of 150 case-patients and twice the number of control participants as case-patients per stratum. In all, 896 potentially eligible children (290 with positive test results and 606 with negative test results for SARS-CoV-2) were identified and telephoned an average of 32 days after SARS-CoV-2 testing. In all, 494 parents or guardians could not be contacted or refused, and five were excluded because the child had been hospitalized with COVID-19, leaving 397 participants, including 154 case-patients (positive SARS-CoV-2 test results) and 243 control participants (negative SARS-CoV-2 test results). Trained interviewers administered structured interviews in English or Spanish (three interviews) by telephone and entered data into REDCap software (*2*). This project was deemed nonresearch public health practice by the CDC and the University of Mississippi Medical Center Institutional Review Boards and conducted consistent with applicable federal law and CDC policy.^¶^

Data collected included participant demographic characteristics, symptoms, close contact (within 6 feet for ≥15 minutes) with a person with known COVID-19, school or child care attendance, and family or community exposures ≤14 days before the SARS-CoV-2 test. For participants who attended in-person school or child care, parents or guardians were asked about the frequency of mask use among students and staff members inside the facility. Parents were also asked about frequency of mask use and social distancing by child and among other persons present for each community exposure. Descriptive and statistical analyses were performed to compare case-patients with control participants, assessing differences in demographic characteristics, school, community exposures, and close contact. Logistic regression models accounting for child sex, age group, and race/ethnicity were used to estimate aORs and 95% CIs, comparing odds of exposures among case-patients and control participants. In each model, SARS-CoV-2 test result (i.e., positive or negative) was the outcome variable, and each exposure response was the predictor variable. Statistical analyses were conducted using SAS software (version 9.4; SAS Institute).

Among the 397 participants, 82 (21%) were aged <4 years, 214 (54%) were female, 217 (55%) were non-Hispanic Black, and 145 (37%) were non-Hispanic White (Table). Participants were tested in outpatient health facilities (78%) or emergency departments (22%); 53% were tested because they were experiencing symptoms; case-patients were more likely than were control participants to be tested because of close contact with a COVID-19 case (66% versus 41%) (p<0.01). Overall, case-patients were more likely to have had close contact with a person with known COVID-19 than control participants (aOR = 3.2, 95% CI = 2.0–5.0); 64% of close contacts of case-patients and 48% of those of control participants were family members (p = 0.02), whereas school or child care classmates were reported as close contacts for 15% and 27%, respectively (p = 0.04). In-person school or child care attendance ≤14 days before the SARS-CoV-2 test was reported for 62% of case-patients and 68% of control participants and was not associated with a positive SARS-CoV-2 test result (aOR = 0.8, 95% CI = 0.5–1.3). Among 236 children aged ≥2 years who attended child care or school during the 2 weeks before the SARS-CoV-2 test, parents of 64% of case-patients and 76% of control participants reported that their child and all staff members wore masks inside the facility (aOR = 0.4, 95% CI = 0.2–0.8).

**TABLE:** Characteristics of children and adolescents aged <18 years who received positive and negative SARS-CoV-2 test results (N = 397)* — Mississippi, September–November 2020

Characteristic	No. (%)		P-value^†^
	Case-patients	Control-participants	
	(n = 154)	(n = 243)	
**Age group, yrs**			0.17
<4	38 (25)	44 (18)	
4–8	28 (18)	62 (26)	
9–14	60 (39)	101 (42)	
15–17	28 (18)	36 (15)	
**Sex**			0.32
Male	68 (44)	115 (47)	
Female	86 (56)	128 (53)	
**Race/Ethnicity (missing = 20)**			0.15
Black, non-Hispanic	92 (62)	125 (55)	
Hispanic	4 (3)	2 (1)	
Other, non-Hispanic	2 (1)	7 (3)	
White, non-Hispanic	50 (34)	95 (41)	
**Clinical setting**			0.97
Emergency department	34 (22)	54 (22)	
Outpatient	120 (78)	189 (78)	
**Reason for SARS-CoV-2 testing^§^**			
Felt unwell	86 (56)	123 (51)	0.31
Close contact with COVID-19 case	101 (66)	99 (41)	<0.01
Required for school/day care	1 (1)	14 (6)	0.01
**Previous close contact with a person with known COVID-19 (missing = 10)**	104 (69)	100 (42)	<0.01
**Relationship to close contact with known COVID-19^§^ (n = 204)**			
Family member	67 (64)	48 (48)	0.02
Friend	8 (8)	15 (15)	0.10
School classmate	16 (15)	27 (27)	0.04
**Household size, mean (SD)**	4.5 (1.3)	4.4 (1.5)	0.21
**Residence type (missing = 11)**			0.37
Single family home	119 (78)	196 (84)	
Apartment building	28 (18)	31 (13)	
Group home	5 (3)	7 (3)	
**School or child care exposure ≤14 days before SARS-CoV-2 test^¶^ (missing = 7)**			0.24
In classroom or child care	95 (62)	161 (68)	
At home	58 (38)	76 (32)	
**Among participants attending school or child care (n = 256)^¶^**			
Days per week, mean	4.6 (0.9)	4.5 (1.0)	0.24
Hybrid model with some days at home	18 (19)	36 (23)	0.46
>10 students per classroom	60 (76)	96 (72)	0.45
Indoor school activities	17 (19)	29 (19)	1.00
Community exposure ≤14 days before SARS-CoV-2 test**			
Social gatherings	17 (11)	13 (6)	0.04
Sporting events or concerts	26 (18)	46 (20)	0.62
Religious services	19 (13)	42 (18)	0.16
Child gatherings (e.g., birthday parties, playdates)	14 (9)	9 (4)	0.03
Travel with others	8 (5)	7 (3)	0.26
Visitors in home	61 (42)	72 (31)	0.05
Restaurants	29 (20)	37 (16)	0.35
Household member working in health care with patient contact	36 (24)	50 (21)	0.62

**Abbreviations:** COVID-19 = coronavirus disease 2019; SD = standard deviation.

* Respondents who completed the interview and average of 32 days after their child’s test date.

^†^ P-value for comparison of characteristics of case-patients with control participants using Fisher’s exact text or Pearson’s chi-squared test for categorical variables or Wilcoxon rank sum test for continuous variables.

^§^ Parents could provide more than one response.

^¶^ Questions about school attendance and participation in athletics or school-related activities were “Did your child attend school in-person (all of the week, part of the week [part time virtual], none of the week [all virtual])” (missing = 6); “How many days per week did your child attend daycare/school outside the home?”; “On the days when the child attended daycare/school in person, was the classroom (less than half full (<5 students), more than half full (5-10 students), full (approximately 10 students), more than full (>10 students); responses were dichotomized as more than full (yes/no)”; “Did your child participate in any indoor school-related activities like choir, band, clubs, etc.?” (missing = 9); “Did your child participate in any indoor sports like basketball, volleyball, etc.?” (missing = 4). Attending school or child care was dichotomized as ≥1 day in the past 2 weeks or none. For affirmative responses about the child’s participation in sports or school-related activities, parents were asked to specify activities.

** Community exposure questions asked in reference to the 2 weeks before the child’s SARS-CoV-2 test were “Did your family/household attend any social gatherings with other people who do not live in your home (like weddings, funerals, parties, celebrations, etc.)?” (missing = 15); “Did your family/members of your household attend any sporting events or concerts?” (missing = 14); “Did your family/household attend meetings or religious services with 10 or more people who do not live with you?” (missing = 12); “Did your child attend any gatherings (10 or more children) outside of the home or school (like birthday parties, playdates, etc.)?” (missing = 14); “Did your family/household travel with any other people/families who do not live with you?” (missing = 10); “Did you receive visitors into your home?” (missing = 21); “Did your family/household eat in restaurants?” (missing = 21); “Are you or anyone in the household a health care provider that provides direct patient contact?” (missing = 10). For each affirmative response, respondents were asked if the activity took place inside or outside, if other persons at the event were masked (everyone, some, no one), and if social distancing was observed.

Compared with control participants, case-patients were more likely to have attended gatherings with persons outside their household, including social functions (aOR = 2.4, 95% CI = 1.1–5.5), activities with children (aOR = 3.3, 95% CI = 1.3–8.4), or to have had visitors at home (aOR = 1.9, 95% CI = 1.2–2.9) during the 14 days before the SARS-CoV-2 test (Figure); 27% of all parents whose children attended social gatherings reported mask use by all persons present and 46% reported adherence to social distancing, whereas 16% and 39%, respectively reported mask use and social distancing when having visitors in the home.

**FIGURE:**
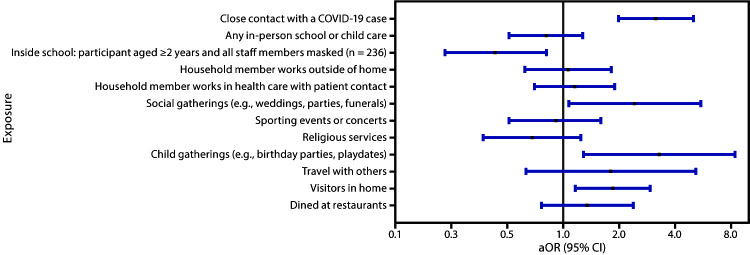
Adjusted odds ratios (aORs)* and 95% confidence intervals (CIs) for close contact, school or child care, and community exposures^†^ associated with confirmed COVID-19 among children and adolescents aged <18 years (N = 397) — Mississippi, September–November 2020 **Abbreviation:** COVID-19 = coronavirus disease 2019. * Odds ratios were estimated using logistic regression models adjusting for sex, age group, and race/ethnicity. ^†^ Close contact, school or child care, and community exposure questions asked in reference to the 2 weeks before the child’s SARS-CoV-2 test were “Did the child have close contact with another person with confirmed COVID-19?”; “Did your child attend school in person (all of the week, part of the week [part time virtual], none of the week [all virtual])” (missing = 6); "Did your child wear a mask inside at daycare/school? (all the time, some of the time, none of the time)?" (missing = 15); "Did the teachers/staff at your child's daycare/school wear a mask inside (all of the time, some of the time, none of the time)?" (missing = 15); “Did your family/household attend any social gatherings with other people who do not live in your home (like weddings, funerals, parties, celebrations, etc.)?” (missing = 13); “Did your family/members of your household attend any sporting events or concerts?” (missing = 12); “Did your family/household attend meetings or religious services with 10 or more people who do not live with you?” (missing = 11); “Did your child attend any gatherings (10 or more children) outside of the home or school (like birthday parties, playdates, etc.)?” (missing = 12); “Did your family/household travel with any other people/families who do not live with you?” (missing = 8); “Did you receive visitors into your home?” (missing = 19); “Did your family/household eat in restaurants?” (missing = 19); “Are you or anyone in the household a health care provider that provides direct patient contact?” (missing = 8). For each affirmative response, respondents were asked if the activity took place inside or outside, if other persons at the event were masked (everyone, some, or no one) and if social distancing was observed. Mask use inside school by the child and all staff members was dichotomized as all the time (for both questions) versus all other responses.

## Discussion

In this investigation, children and adolescents who received positive test results for SARS-CoV-2 were more likely than were similarly aged participants who had negative test results to have had reported close contact with a person with confirmed COVID-19 and less likely to have had reported consistent mask use by students and staff members inside the school facility. Among participants with close contact with a person with COVID-19, close contacts of case-patients were more likely to be family members and less likely to be school or child care classmates than were those of control participants. Attending in-person school or child care during the 2 weeks before the SARS-CoV-2 test was not associated with increased likelihood of a positive SARS-CoV-2 test result. The majority of respondents reported universal mask use inside school and child care facilities as recommended by Mississippi State Department of Health,** although parents of case-patients were less likely than were those of control participants to report consistent mask use indoors among their child aged ≥2 years and staff members. Efforts to reduce COVID-19 in families and communities, in addition to mitigation strategies in schools and child care programs, are important for preventing transmission to children and adolescents.^††^ With increasing COVID-19 incidence and various behaviors across the country, timely investigations to identify activities associated with SARS-CoV-2 transmission can inform targeted mitigation strategies at local levels.

Among children and adolescents with COVID-19, 69% reported close contact with a person with COVID-19, similar to previous findings among children and adults (*3*–*5*). Most close contact exposures were to family members, consistent with household transmission of SARS-CoV-2 (*6*–*8*). Fewer (42%) children who received a negative SARS-CoV-2 test result reported close contact with a person with known COVID-19. To help slow the spread of SARS-CoV-2, persons exposed to someone with COVID-19 should stay home, in addition to adhering to recommendations to wear masks, maintain social distance, and wash hands often.^§§^ If a family member or other close contact is ill, additional prevention measures can be taken to reduce transmission, such as wearing masks, reducing shared meals and items, cleaning and disinfecting the home, and wearing gloves for those with and without known COVID-19.^¶¶^

The findings in this report are subject to at least four limitations. First, the sample included 397 children and adolescents tested during September–November 2020 at health care facilities associated with one large academic medical center in Mississippi and might not be representative of children and adolescents in other geographic areas of the United States. Further, parents of eligible children who could not be contacted or refused to participate could be systematically different from those who were interviewed for this investigation. Second, unmeasured confounding is possible, such that reported behaviors might represent factors, including concurrently participating in activities in which possible exposures could have taken place, that were not included in the analysis or measured in the study. Most respondents were aware of their child’s SARS-CoV-2 test results and interviews were conducted several weeks after testing, factors which could have influenced parent responses. Third, parent report of frequency of mask or cloth face covering use at schools and child care programs was not verified. Finally, case or control status might be subject to misclassification because of imperfect sensitivity or specificity of PCR-based testing.

This investigation highlights differences in community and close contact exposures and in-school mask use between children and adolescents who received a positive SARS-CoV-2 test result and those who received a negative SARS-CoV-2 test result during the beginning of the 2020–21 academic year in Mississippi. Continued efforts to prevent transmission at schools and child care programs are important, as are assessments of various types of activities and exposures to identify risk factors for COVID-19 as children engage in classroom and social interactions (*9*,*10*). Exposures and activities in which persons are less likely to maintain mask use and social distancing, including family gatherings and group activities, might be important risk factors for SARS-CoV-2 infection among children and adolescents. Promoting behaviors to reduce exposures to SARS-CoV-2 among children and adolescents in the household and community, as well as in schools and child care programs, is needed to prevent COVID-19 outbreaks at schools*** and child care programs and slow the spread of COVID-19.

**Summary**

**What is already known about the topic?**

Community and close contact exposures contribute to the spread of COVID-19.

**What is added by this report?**

Among children and adolescents aged <18 years in Mississippi, close contact with persons with COVID-19 and gatherings with persons outside the household and lack of consistent mask use in school were associated with SARS-CoV-2 infection, whereas attending school or child care was not associated with receiving positive SARS-CoV-2 test results.

**What are the implications for public health practice?**

Close contacts with persons with COVID-19 and gatherings contribute to SARS-CoV-2 infections in children and adolescents. Consistent use of face masks and social distancing continue to be important to prevent COVID-19 spread.

## Footnotes

* https://covid.cdc.gov/covid-data-tracker/#demographics. Aged <18 years = 1,207,363 (10.2%) of 11,886,368 cases with age group available.

† Respondents were asked “Did your family/household attend any social gatherings with other persons who do not live in your home (like weddings, funerals, parties, celebrations, etc.)?” and “Did your child attend any gatherings (10 or more children) outside of the home or school (like birthday parties, playdates, etc.)?”

§ Overlapping dates resulted from the approach used to create the three lists; duplicates were removed.

¶ Activity was determined to meet the requirements of public health surveillance as defined in 45 CFR 46.102(l)(2).

**
https://msdh.ms.gov/msdhsite/_static/resources/9917.pdf .

††
https://www.cdc.gov/coronavirus/2019-ncov/community/schools-childcare/index.html .

§§
https://www.cdc.gov/coronavirus/2019-ncov/prevent-getting-sick/disinfecting-your-home.html .

¶¶ https://www.cdc.gov/coronavirus/2019-ncov/if-you-are-sick/index.html; https://www.cdc.gov/coronavirus/2019-ncov/if-you-are-sick/quarantine.html.

***
https://www.msdh.ms.gov/msdhsite/_static/resources/11292.pdf .
